# Lessons learned on recruitment and retention in hard-to-reach families in a phase III randomised controlled trial of preparatory information for children undergoing general anaesthesia

**DOI:** 10.1186/s12903-017-0411-4

**Published:** 2017-09-07

**Authors:** C. Huntington, J. Timothy Newton, N. Donaldson, C. Liossi, P. A. Reynolds, R. Alharatani, M. T. Hosey

**Affiliations:** 1 King’s College London Dental Institute, Bessemer Road, London, SE5 9RS UK; 2 University of Southampton and Great Ormond Street Hospital for Children NHS Trust, Southampton, UK

**Keywords:** Paediatric clinical trials, Recruitment, Retention, Hard to reach families, Dentistry, General Anaesthesia

## Abstract

**Background:**

Recruitment and retention are documented as two of the most difficult elements of conducting clinical trials. These issues are even more challenging in paediatric trials, particularly when the families being recruited and retained are deemed ‘hard to reach’.

**Methods:**

Through the authors’ own reflection on the conduct of the trial this paper examines recruitment and retention with hard to reach families from the perspective of a recently completed clinical trial on preparatory information for children undergoing general anaesthesia for tooth extractions in which approximately 83% of those approached and eligible agreed to participate.

**Results:**

The lessons learned for recruitment include: the importance of children’s assent; maximising limited resources when screening and approaching potential participants; valuing families’ time; and developing effective professional relationships. The retention rate was 83-85.5% at follow up time points up to 3.5 weeks following recruitment, insights into how this was accomplished include: ensuring continuity of care; determination to connect via telephone; valuing families’ time; and close monitoring of appointment date changes.

**Conclusions:**

Implications for future paediatric trials with hard to reach families are discussed.

**Trial registration:**

ISRCTN18265148; NIHR Portfolio 10,006. Date of Registration: 29 November 2013. The trial was registered after commencement but before completion of data collection.

## Background

Recruitment and retention are often perceived as challenges to conducting clinical trials [1–4]. Many trials are unable to recruit sufficiently to meet the requirements of the a priori power calculation, and close prematurely or require an extension [3–6]. Several reviews including a Cochrane systematic review, state that little is known about successful recruitment to RCTs [2, 6–9]. Recruitment to paediatric trials in particular, has a history of low participation and faces specific challenges including the ethical issues of recruiting children [7, 10–14]. More paediatric clinical trials are required to ensure that children and families are receiving acceptable care and gaining access to new opportunities in health [8], so it is important that strategies for successful recruitment and retention are examined. The difficulties of recruiting and retaining families can be further heightened by the challenges that disadvantaged families face [15–19]. These families - often termed ‘hard to reach’ - are less likely to access services, despite needing them more than others and they would particularly benefit from improved interventions [18–25]. They are also less likely to participate in studies due to both logistical and attitudinal barriers [16–18, 20, 26]. It is likely that without the inclusion of such families, the generalisability of any findings is limited [17, 19, 24, 25].

A separate but related issue concerns retention of participants within a trial. Retention rates in clinical trials tend to be poorly reported [26] but previous studies have indicated that attrition can be as much as 70%, which is likely to have a significant impact on the generaliasability of any study findings [24, 26, 27].

Previous research indicates that strategies to facilitate recruitment and retention include: staff working out of hours; face to face recruiting; recruiter(s) developing a relationship with recruitment site staff, recruiters having strong interpersonal skills, good engagement with each participant equally to help them learn about the health issue being addressed; ensuring little or no extra effort is required on the part of participants; conducting a pilot study, and discussing the advantages and disadvantages of participation [4, 9, 24, 28–31]. Financial incentives have previously been reported to improve participation [6, 18, 19, 29, 32], but this is not common practice and raises the ethical issue of the possibility of coercion [4].

The purpose of this paper is to present the lessons learned from recruitment and retention of families to a clinical trial of preparatory information for children undergoing general anaesthesia (GA) for tooth extraction [33].

## Methods

The Phase III RCT [33] recruited families whose five to seven year old children were undergoing GA because a child in the family needed teeth extracted due to serious decay. A recent UK audit of GA service provision for dental surgery has shown the national yearly dental case load total of 111,600, of which 60% were children [34]. For full details of the trial methodology see Hosey et al. [33]. Families with a child eligible to enter the trial were referred by primary care dentists to the hospital based specialist paediatric dentist service. The aim of the RCT was to understand whether a preparatory computer package accessed from home and based on cognitive behavioural principles influenced children’s ability to cope with the GA induction and various other elements of the experience. Parents were asked questions regarding their children’s overall behaviour. Children were asked about their anxiety levels and their feelings about going to the dentist and then observed throughout their time in the hospital, particularly the child’s behaviour before, during and after anaesthetic induction. These families were then asked about the hospital experience, whether the preparatory information assisted the families, and about physical and psychological morbidity following the procedure. There were four data collection time points:

1. Recruitment occurred during the families pre-GA assessment appointment 2 weeks prior to GA
2. The child’s GA appointment
3. A telephone call 48 h post GA
4. A telephone call one week post GA

Initially, it was planned that this study would take 18 months to complete recruitment and, despite some obstacles (including cancelled theatre lists that were essential to collecting trial data), the recruitment target was obtained in 17 months. Recruitment was conducted in a small waiting room outside a nurse led medical pre-assessment clinic for children who were having GA. This occurred 1 day a week from 7.45 until 17.30 within the hospital day surgery unit. Our target children, those whose dental health was so severe as to necessitate having teeth extracted under dental GA, made up between half and three-quarters of all families who attended each clinic. Children attending the service, both locally and nationally, are typically 6 years of age and have an average of seven primary teeth removed. The dental diagnosis of this level of tooth decay is known as Early Childhood Caries. The Association of Paediatric Anaesthetists of Great Britain and Ireland Guidelines [35] have recommended that these children have access to preoperative preparation. When considering recruitment and retention in this trial, it is important to acknowledge the connection between early childhood caries and socio-economic deprivation [25, 36–40]. The families referred for this treatment have high levels of caries, and the catchment area includes some of the worst areas of social deprivation in London. Indeed, more than a third of children in the catchment area would be considered to be living in deprivation [37].

### Pilot study

A pilot study was conducted which sought to: (i) estimate the recruitment rate by confirming inclusion and exclusion criteria and willingness of potential recruits to take part; (ii) calibrate the blinded observer’s scoring of the Visual Analogue Scale (VAS) at the time of GA induction and train in taking the other measures such as the modified Yale Pre-operative Anxiety Scale (mY-PAS); (iii) determine the best way to enable blinding both of the participants and the blind researcher on the ward; and (iv) avoidance of the ‘Hawthorne effect’; (v) test the randomisation process; (vi) train in video camera data capture The pilot study followed the same design and recruitment criteria as the main RCT, the only difference was that children were only assigned to one group. In order to mimic the actual protocol of the main study, participating children were given a two-sided colouring sheet at the pre-assessment clinic to take home. Their compliance was checked pre-operatively on the day of the surgery, by asking the children if they had coloured the pages.

### Sample description – Main trial

A total of 319 families were approached with 134 excluded or declining - 95 were excluded during the initial questioning to confirm eligibility and 39 declined to participate. Table 1 provides further details on reasons that different families were excluded or declined to participate. With regard to attendance at the clinic, 221 children attended with their mothers, 64 children with their fathers and 27 children attended with both parents.

**Table 1:** Reasons that families were excluded or declined following being approached

Found not to meet criteria	95	Declined to participate	39
Non-English speaking	48	Too busy	12
		Declined without reason	7
Child had previous GA	14	Parent wanted to think about it	5
Attended with adult who could not legally consent	7	Child did not give assent	2
Had another hospital appointment	2	Left while recruiter was with another family	3
No computer at home	1	Parent did not want child to know	3
Safeguarding concerns	1	Other	7

A total of 185 families were successfully recruited. The recruited children’s mean age was 6.0 years old which is similar to previous research that states the mean/median age of GA for early childhood caries is five to seven years old [38, 41–43] with the distribution of gender being similar to previous studies (49.3% girls) [42, 44]. At baseline, 31% of the sample reached the level of possible clinically significant psychological disturbance (scores above 11 on the Revised Rutter for School Age Children) [45]. It is interesting to note that input from the Patient and Public Involvement initiative (PPI) which informed the trial design, suggested that video recording children while the child was being put to sleep would not present an issue; however during the actual recruitment, 22 families indicated that they were unwilling to consent to their child being video recorded.

Based on the successful recruitment, the authors reflected on those strategies that they felt had been most effective in ensuring participation. These qualitative reflections were combined with an analysis of the published literature on enhancing recruitment and retention in clinical trials to produce a personal analysis of the lessons learned from this trial. Since there was no formal manipulation of strategies or determination of the effect of different strategies to determine the effect on response rates, the lessons learnt will be described within the context of a discussion of the existing literature.

## Results and discussion

Five lessons were learned for promoting recruitment, as follows:

- The Importance of Children’s Assent
- Maximising Limited Resources when Screening and Approaching Potential Participants
- Valuing Family’s Time
- Developing Effective Professional Relationships
- Flexibility with Recruitment Timings

### The Importance of Children’s Assent

Ensuring that the children themselves understand what the research involves, is considered good clinical and ethical practice [46, 47]. The value of this can be overlooked if attention is overly focused on parental consent [48, 49]. We found that time spent ensuring the children themselves were willing to take part made a difference to family engagement. Many paediatric researchers discuss assent and its importance in clinical trials, particularly if children are able to understand [48–51]. For example, Alderson explains that consent involves competence, respect, dignity, informed choice and understanding [47]. She states that whilst this is possible with children, it is more time consuming and requires more consideration than with adults, however there are tangible benefits to be gained.

One strategy for enabling children’s understanding was a specific information sheet with pictures and a low Gunning Fog Index. Children were encouraged to read through this sheet themselves and if unable, the parent or researcher helped the child. We found that asking the parent *and* the child if either had questions about the study ensured that both understood the project and were willing to participate. Additionally, a separate assent form for children was provided, as stipulated by the UK NHS ethics committee process. Whenever children were unable to write their name and their parents completed it for them, the researcher ensured that the child understood all parts and willingly answered the questions. Furthermore, all study measures that invited children’s responses were augmented with facial images. This meant that the child could point to individual faces to indicate their feelings instead of having to verbally articulate it [52].

Two children chose not to participate after having the project explained to them, despite their parents’ willingness to participate. The child’s reasons for this were unclear.

#### Maximising Resources when Screening and Approaching Potential Participants

During the pilot study for the project, it was found that a high number of ineligible families were approached and agreed to participate and then had to be later excluded. Ninety-seven participants were approached of whom 41 agreed to participate (42%). Twenty participants were excluded for reasons relating to the inclusion criteria. This proved challenging, particularly when exclusion was due to a child experiencing learning difficulties. Therefore, before the recruitment clinic the recruiter would obtain the list of all children who were scheduled to attend the pre-GA clinic that week. Their patient files would then be scrutinised to identify which children would not meet the inclusion criteria. Any families who may meet the criteria were approached. While this was time-consuming prior to recruitment, it proved an invaluable process that led to appropriate usage of the trial’s allocated resources.

Furthermore, the job plan and working hours of the Research Staff employed for the project was set up specifically to be flexible in order to maximise opportunities for recruitment and data collection. Matching the working hours of the research staff to the clinics was felt to have been very important in maximising recruitment and retention.

### Valuing the time given by families

One reason that families reported being unable to participate is that they were “too busy” and many parents approached asked about the length of time needed if they agreed to take part. One way that this was handled was to offer the family to take some questionnaire measures home and return them on their next visit. This occurred in less than 10% of cases but was offered to over half the families. Simply offering parents this option seemed to make them feel more open to participating, possibly by demonstrating that the researchers would be sensitive to their time constraints and needs.

In other cases, the recruiter spent longer with families if they had a great deal of questions or wanted to discuss the project in more detail. Previous research reports that being able to spend time with potential participants made them more likely to agree to take part [53]. Thus families felt respected and the researcher facilitated their participation, by demonstrating a flexible approach open to family needs.

### Developing Effective Professional Relationships

One of the most important elements for recruitment was developing effective relationships with hospital staff, including the nursing staff, administrative team and the hospital paediatric dentists. As part of the pilot work the recruiter learned the processes of the day surgery unit and staff. For the smooth running of the RCT, it was vital to reassure the nurses in the clinic where recruitment occurred that the research was feasible and did not pose a risk to either the children or the clinic’s operations or interfere with the throughput of the clinic. The research staff involved in recruitment asked these stakeholders for their perspectives on intervention and the study processes were shaped to fit the service incorporated during the pilot phase of the process. The nurses and recruiter also liaised closely to ensure that the family didn’t miss their allotted nurse assessment, even if this meant that the recruiter allowed the data collection to be intercepted. Then the recruiter re-engaged with the family following the nurses’ assessment. This ensured that fluid pathways were created between the day surgery staff, the recruiter and the families. Through this process, these stakeholders had an active role in supporting the trial and may have increased the likelihood of family participation because some of the families are likely to have trusted the nurses as sources.

### Flexibility with Recruitment Timings

As mentioned previously, the research staff adopted a flexible approach to the timing of data collection and spent full days in the clinic just waiting for potential participants to attend the hospital appointment. During the pilot study, ten children had been missed because it was thought that they would arrive near their scheduled appointment time, but some potential participants arrived as much as 2 h before or 2 h afterwards. The nurses who carried out the medical physical checks were willing to see the families whenever they arrived and so the recruiter had to do likewise. Obviously, this was only possible due to having a dedicated recruiter, and in this case, this proved to be essential to capturing potential families.

## Lessons learned concerning retention of participants

As with all clinical trials, retention proved challenging. In this RCT, participants were asked to participate in a telephone interview both 48 h and 1 week after their child’s GA visit. This was to assess physical morbidity, psychological morbidity and family satisfaction with the dental general anaesthesia treatment and also with the preparatory information that was provided. During the pilot study, parents were asked to choose the time they would prefer to be telephoned and most responded that “any time” was suitable. However, the response rate at 48 h was 56% and, at 1 week only 58.5% successfully completed the telephone call interview. Although most studies deem 50–80%, is acceptable [53, 54], the current RCT had hoped for better. The Centre for Evidence Based Medicine at Oxford University uses 20% attrition to define trial quality [55].

Therefore, the goal for this RCT was a 80% or higher success rate at follow-up. As such, two key strategies were implemented:

1. Parents were given a telephone appointment card that gave the name of the caller, and the date and time at which they would be called. Even if parents responded that they could be called “any time” they were are asked to “pin down” a specific date and time.
2. Parents were alerted to the fact that the call would come from a ‘withheld’ number because it came from a dedicated hospital telephone. Parents in the Pilot Study had reported that a reason for not answering their telephones was because the number was not recognised and so, were pleased to learn that the call would come from a withheld number at a pre-arranged time.

These two strategies improved response rates considerably: data were collected at the 48 h telephone call by 151 of the 176 participants (a 85.5% success rate) and the 1 week telephone call had a 83.1% success rate (146 participants contributed data). Using telephone calls for data collection following a hospital event, has the advantage of enabling multiple attempts to make contact and well as giving the family flexibility in timing the delivery of their response. Some studies have used home visits to achieve this and have also reported high retention rates. One such study reported a 87.5% retention rate at the 48 h home visit [56] while another reported a 96.1% retention rate at a 48 h home visit [57]. Although arrangements for home visits are likely to be more difficult, particularly in socially deprived neighbourhoods, they do seem to result in high rates of retention. Perhaps this ‘personal’ approach is highly valued by these families? Conversely, other studies have suggested that offering the follow up telephone calls rather than a hospital or home visit may have indirectly facilitate recruitment because it did not cause inconvenience [9].

Surprisingly, a non-response at the telephone follow-up interview was not related to any demographic variable. In particular: Chi-square analyses exploring the relationship between response to the follow up interview and demographic characteristics revealed that having a mother at home (all participants lived with their mothers) or father at home (*p* = 0.24); and it was not related to the number of children in the family (*p* = 0.42). Moreover, the number of teeth that were extracted did not influence a lack of response, either at home (*p* = 0.40) or immediately post-operatively (*p* = 0.10). The parental education level also had no influence (*p* = 0.93).

The following variables were found to enhance retention rates:

- Continuity of care
- Repeated attempts to connect via the telephone
- Valuing families’ time
- Tracking hospital appointment date changes

### Continuity of care

An additional component that may have supported the retention of families was the continuity of contact with the research team. Throughout the typical NHS hospital process, the families rarely see the same hospital worker. However, in this study, the same person who recruited the families onto the trial, also saw them on the day of the child’s GA, and then completed the follow up calls. Although the recruiter aimed to be as inconspicuous as possible, by the fourth discussion (at the 1 week phone call), many of the families seemed relaxed enough to speak honestly and openly.

### Repeated attempts to connect via the telephone were made

In order to achieve the 142/184 successfully completed telephone calls at the 48 h data collection time-point, more than 500 calls were made. More than 650 telephone calls were made to collect 138/184 at 1 week. These were conducted between 8.00 until 19.30 to families to answer the calls either before work or in the evening.

### Valuing families’ time

As with recruitment, valuing the families’ time was essential to retention. When each family answered a telephone call, the first question they were asked was “is this a good time?”. If the family said “no”, the researcher always offered to phone the family at another, more convenient time. This demonstrated that the researchers’ respect for the family while also encouraging the parent to answer when they were called back. This approach demonstrates the ‘continuing consent’ process, which is considered to be good practice [48, 58].

### Tracking hospital appointment date changes

Occasionally, a child’s hospital general anaesthetic day surgery date is rescheduled, e.g. if the child has an upper respiratory tract infection (a ‘cold’). In this phase III RCT, the researcher (CH) continually monitored the operation lists; had she not done so, 14/184 families would have been missed.

Considerations of these reflections on the response rate for this study must be tempered by an analysis of the limitations of this study. Primary amongst this is the fact that this study did not seek to systematically determine the effect of recruitment strategies on recruitment and retention. Without such experimental data it is impossible to determine whether the high rates of recruitment and retention reported here are truly the result of the strategies identified by the researchers or of some other factor. Further research, perhaps using embedded variation of recruitment and retention strategies within larger clinical trials could explore this. Secondly while the validity of the inferences drawn by the researchers were checked against the existing literature, the analysis remains a reflection of the views of those directly involved in the project – a more independent analysis may have revealed alternative interpretations.

## Conclusions

The excellent recruitment and retention rates found in this phase III RCT were the result of strategies that were identified both from the previous literature and developed in the pilot study. The participating families are ‘hard-to-reach’ and were drawn from areas of socio-economic deprivation and have been traditionally characterised as having poor engagement with clinical services, let alone research. Underpinning many of the strategies identified in this study is the importance of positive communication and in the relationship between the researchers and the participants [1, 24]. However, there is a potential for conflict here, in that whilst a good relationship might increase recruitment and retention, it might also increase the likelihood of a Hawthorne effect. Many of these families might value the personal approach that participation in a study such as this will offer.

Future studies might report on how much each of the aforementioned strategies contribute to families’ ongoing participation and retention by embedding studies of recruitment strategies within the larger clinical trial.

## References

[1] Fletcher B, Gheorghe A, Moore D, Wilson S, Damery S (2012). Improving the recruitment activity of clinicians in randomised controlled trials: a systematic review. BMJ Open.

[2] Foy R, Parry J, Duggan A, Delaney B, Wilson S, Lewin-van den Broek NT (2003). How evidence based are recruitment strategies to randomized controlled trials in primary care? Experience from seven studies. Fam Practice.

[3] Friedman LM, Furberg CD, DeMets DL (2010). Fundamentals of clinical trials.

[4] Kaur G, Smyth RL, Williamson P (2012). Developing a survey of barriers and facilitators to recruitment in randomized controlled trials. Trials.

[5] Blanton S, Morris DM, Prettyman MG, McCulloch K, Redmond S, Light KE (2006). Lessons learned in participant recruitment and retention: the EXCITE trial. Physic Therap.

[6] Treweek S, Lockhart P, Pitkethly M, Cook JA, Kjeldstrom M, Johansen M (2013). Methods to improve recruitment to randomised control trials: Cochrane systematic review and meta-analysis. BMJ Open.

[7] Smyth RL, Weindling AM (1999). Research in children: ethical and scientific aspects. Paediatr.

[8] Hargreaves DS (2012). The Lancet’s support for clinical trials in children and young people. Lancet.

[9] Caldwell PHY, Hamilton S, Tan A, Craig JC (2010). Strategies for increasing recruitment to randomised controlled trials: systematic review. PLoS Med.

[10] Afshar K, Lodha A, Costei A, Vaneyke N (2005). Recruitment in pediatric clinical trials: an ethical perspective. J Urology.

[11] Field MJ, Berman RE (2004). Ethical conduct of clinical research involving children.

[12] Knox CA, Burkhart PV (2007). Issues relating to children participating in clinical research. J Pediatr Nurs.

[13] Caldwell PHY, Murphy SB, Butow PN, Craig JC (2004). Clinical trials in children. Lancet..

[14] Bartlett C, Doyal L, Ebrahim S, Davey P, Bachmann M, Egger M (2005). The causes and effects of socio-demographic exclusions from clinical trials. Health Tech Assess.

[15] Baxter J, Vehik K, Johnson SB, Lernmark B, Roth R, Simell T (2012). Differences in recruitment and early retention among ethnic minority participants in a large pediatric cohort: the TEDDY study. Contemp Clin Trials.

[16] Patel MX, Doku V, Tennekon L (2003). Challenges in recruitment of research participants. Adv Psych Treat.

[17] UyBico SJ, Pavel S, Gross CP (2007). Recruiting vulnerable populations in research: a systematic review of recruitment interventions. Soc Gen Inter Med.

[18] Zook PM, Jordan C, Adams B, Visnessa CM, Walter M, Pollenz K (2010). Retention strategies and predictors of attrition in an urban pediatric asthma study. Clin Trials.

[19] Senturia YD, Mortimer KM, Baker D, Gergen P, Mitchell H, Joseph C (1998). Successful techniques for retention of study participants in an inner-city population. Control Clin Trials.

[20] Department for Children, Schools and Families. Outreach to children and families: a scoping study. Research Report DCSF-RR116; 2009.

[21] HM Treasury and Department for Education and Schools. Aiming high for children: Supporting families. ISBN: 978-1-84532-261-8 PU188 Printed by The Stationery Office; 2007.

[22] Katz I, La Placa V, Hunter S (2007). Barriers to inclusion and successful engagement of parents in mainstream services.

[23] Rae WA, Brunnquell D, Sullivan JR, Roberts MC (2003). Ethical and legal issues in pedaitric psychology. Handbook of Pediatric Psychology.

[24] Pipel D, Leavitt FJ, Elizur-Leiberman E (1996). Ethical and cross cultural questions concerning pediatric clinical trials. Control Clin Trials..

[25] Gorin S, Hooper CA, Dyson C, Cabral C (2008). Ethical challenges in conducting research with hard to reach families. Child Abu Rev.

[26] Marcellus L (2004). Are we missing anything? Pursuing research on attrition. Can J Nurs Res.

[27] Chadwick BL, Treasure ET (2005). Primary care research: difficulties recruiting preschool children to clinical trials. Internat J Paed Dent.

[28] Bieri D, Reeve R, Champion GD, Addicoat L, Ziegler J (1990). The faces pain scale for the self-assessment of the severity of pain experienced by children: development, initial validation and preliminary investigation for ratio scale properties. Pain.

[29] Robinson KA, Dennison CR, Wayman DM, Pronovost PJ, Needham DM (2007). Systematic review identifies number of strategies important for retaining participants. J Clin Epidem.

[30] Prinz RJ, Smith EP, Dumas JE, Laughlin JE, White DW, Barron R (2001). Recruitment and retention of participants in prevention trials involving family-based interventions. Am J Prev Med.

[31] Badger F, Werrett J (2005). Room for improvement? Reporting response rates and recruitment in nursing research in the past decade. J Adv Nurs.

[32] Bennett C, Khangura S, Brehaut JC, Graham ID, Moher D, Potter BK (2011). Reporting guidelines for survey research: an analysis of published guidance and reporting practices. PLoS Med.

[33] Hosey MT, Donaldson AN, CHuntington C, Liossi C, Reynolds PA, Alharatani R, Newton JT (2014). Improving access to preparatory information for children undergoing general anaesthesia for tooth extraction and their families: study protocol for a phase III randomized controlled trial. Trials.

[34] Sury MRJ, JHMacG P, Cook TM, Pandit JJ (2016). The state of UK dental Anaesthesia: results from the NAP5 activity survey. A national survey by the 5th National Audit Project of the Royal College of Anaesthetists and the Association of Anaesthetists of Great Britain and Ireland. SAAD Digest.

[35] Association of Paediatric Anaesthetists of Great Britain and Ireland. Guidelines for the management of Children Referred for Dental Extractions under General Anaesthesia. London; 2011.

[36] Flanagan SM, Hancock B (2010). ‘reaching the hard to reach’ - lessons learned from the VCS (voluntary and community sector). A qualitative study. BMC Health Serv Res.

[37] Office of National Statistics (2005). Health statistics quarterly, spring.

[38] Petti S (2010). Why guidelines for early childhood caries prevention could be ineffective amongst children at high risk. J Dent..

[39] Steele J, Lader D (2004). Social factors and oral health in children.

[40] Rogers S. Indices of multiple deprivation: find the poorest places in England. The Guardian 29 March 2011. http://www.theguardian.com/news/datablog/2011/mar/29/indices-multiple-deprivation-poverty-england. Accessed 13 July 2017.

[41] Campbell C, Hosey MT, McHugh S (2005). Facilitating coping behaviour in children prior to dental general anaesthesia: a randomized controlled trial. Ped Anesthes.

[42] Hosey MT, Bryce J, Harris P, McHugh S, Campbell C (2006). The behaviour, social status, and number of teeth extracted in children under general anaesthesia: a referral centre revisited. Br Dent J.

[43] Hosey MT, Macpherson LMD, Adair P, Tochel C, Burnside G, Pine C (2006). Dental anxiety, distress at induction and postoperative morbidity in children undergoing tooth extraction using general anaesthesia. Br Dent J.

[44] Olley RC, Hosey MT, Renton T, Gallagher J. Why are children still having preventable extractions under general anaesthetic? A service evaluation of the views of parents of a high caries risk group of children. BDJ. 2011;210(8):E13. 10.1038/sj.bdj.2011.313 21508990

[45] Rutter M (1993). Rutter scales. Child psychology portfolio.

[46] National Institute for Health Research Clinical Research Network (2012). Introduction to good clinical practice (GCP): a practical guide to ethical and scientific quality standards in clinical research.

[47] Alderson P (2007). Competent children? Minors’ consent to health care treatment and research. Soc Sci Med.

[48] Kodish E (2003). Informed consent in pediatric research: is it really possible?. J Pediatr.

[49] Ungar D, Joffe S, Kodish E (2006). Children are not small adults: documentation of assent for research involving children. J Pediatr.

[50] McInstosh N (2004). Ethical principles of research with children. Curr Paediatr.

[51] Marshman Z, Hall MJ (2008). Oral health research with children. Int J Paediatr Dent.

[52] Buchanan H, Niven N (2002). Validation of a facial image scale to assess child dental anxiety. Int J Paediatr Dent.

[53] Fewtrell MS, Kennedy K, Singhal A, Martin RM, Ness A, Hadders-Algra M (2008). How much loss to follow-up is acceptable in long-term randomised trials and prospective studies?. Arch Dis Child.

[54] Kristman V, Manno M, Cote P (2004). Loss to follow-up in cohort studies: how much is too much?. Euro J Epidem.

[55] Centre for Evidence Based Medicine. University of Oxford. 2014; Retrieved from: http://www.cebm.net/. Accessed 13 July 2017.

[56] Hosey MT, Asbury JA, Bowman AW, Millar K, Martin K, Musiello T, Welbury R (2009). The effect of transmucosal 0.2mg/kg Midazolam premedication on dental anxiety, anaesthetic induction and psychological morbidity in children undergoing general anaesthesia for tooth extraction. Br Dent J.

[57] Harth SC, Thong YH (1995). Parental perceptions and attitudes about informed consent in clinical research involving children. Soc Sci Med.

[58] Millar K, Asbury AJ, Bowman AW, Hosey MT, Musiello T, Welbury RR (2006). The effects of brief sevoflurane-nitrous oxide anaesthesia upon children’s postoperative cognition and behaviour. Anaesthesia.

